# Runx2/miR-3960/miR-2861 Positive Feedback Loop Is Responsible for Osteogenic Transdifferentiation of Vascular Smooth Muscle Cells

**DOI:** 10.1155/2015/624037

**Published:** 2015-06-28

**Authors:** Zhu-Ying Xia, Yin Hu, Ping-Li Xie, Si-Yuan Tang, Xiang-Hang Luo, Er-Yuan Liao, Fei Chen, Hui Xie

**Affiliations:** ^1^Institute of Endocrinology and Metabolism, Second Xiang-Ya Hospital of Central South University, Changsha, Hunan 410011, China; ^2^Department of Forensic Medicine, School of Basic Medical Science of Central South University, Changsha, Hunan 410013, China; ^3^School of Nursing of Central South University, Changsha, Hunan 410013, China; ^4^Spine Surgery Department, Second Xiang-Ya Hospital of Central South University, Changsha, Hunan 410011, China

## Abstract

We previously reported that Runx2/miR-3960/miR-2861 regulatory feedback loop stimulates osteoblast differentiation. However, the effect of this feedback loop on the osteogenic transdifferentiation of vascular smooth muscle cells (VSMCs) remains unclear. Our recent study showed that miR-2861 and miR-3960 expression increases significantly during *β*-glycerophosphate-induced osteogenic transdifferentiation of VSMCs. Overexpression of miR-2861 or miR-3960 in VSMCs enhances *β*-glycerophosphate-induced osteoblastogenesis, whereas inhibition of miR-2861 or miR-3960 expression attenuates it. MiR-2861 or miR-3960 promotes osteogenic transdifferentiation of VSMCs by targeting histone deacetylase 5 or Homeobox A2, respectively, resulting in increased runt-related transcription factor 2 (Runx2) protein production. Furthermore, overexpression of Runx2 induces miR-2861 and miR-3960 transcription, and knockdown of Runx2 attenuates *β*-glycerophosphate-induced miR-2861 and miR-3960 transcription in VSMCs. Thus, our data show that Runx2/miR-3960/miR-2861 positive feedback loop plays an important role in osteogenic transdifferentiation of VSMCs and contributes to vascular calcification.

## 1. Introduction

Medial artery calcification is a common and serious problem with ageing of the population. It is a nonocclusive process which leads to decreased vessel elasticity, increased blood pressure, and higher risk of cardiovascular mortality [1–3]. Medial artery calcification is now recognized as a highly regulated process which is similar in many ways to bone mineralization. Osteoblast-like phenotypes transformation of vascular smooth muscular cells (VSMCs), the predominant cells in the tunica media of arteries, is currently considered responsible for the formation of medial artery calcification [4–6]. However, the specific mechanism governing this process is still elusive.

MicroRNAs (miRNAs) are endogenous, small (16~25 nucleotides), single-stranded noncoding RNAs [7]. MicroRNAs mediate translational repression or degradation of target transcript by binding to sites of complementarity in the 3′-UTRs of target mRNAs. During the past decade, they have emerged as powerful posttranscriptional regulators of gene expression [8]. To date, more than 3% of the genes in the human genome have been found to encode for miRNAs, and over 30% of genes in the human genome are estimated to be regulated by miRNAs [9]. It has been documented that miRNAs participate in cellular proliferation, differentiation, migration, and apoptosis [10–12]. However, their roles in osteogenic transdifferentiation or calcification of VSMC have just begun to be understood.

Recently, we reported that Runx2/miR-3960/miR-2861 positive feedback loop is responsible for osteoblast differentiation [13]. Osteoblast differentiation signals lead to the activation of Runx2 transcription factor in stromal cells. In addition to induction of genes essential for osteoblast differentiation, Runx2 transactivates miR-3960/miR-2861. In turn, miR-3960 and miR-2861 maintain the levels of* Runx2 *mRNA and protein via repressing Homeobox A2 (Hoxa2) and histone deacetylase 5 (HDAC5) and stabilizing the osteoblast differentiation. The objective of this study was to elucidate the effect of Runx2/miR-3960/miR-2861 feedback loop on regulation of the osteogenic transdifferentiation of VSMCs.

## 2. Materials and Methods

### 2.1. Cell Culture and Transfection

To isolate primary cells, eight-week-old C57BL/6 male wild type mice received a 150 mg/kg intraperitoneal dose of pentobarbital sodium prior to euthanasia which was confirmed by the absence of a heartbeat. Vascular smooth muscle cells (VSMCs) were isolated from aorta as previously described [14]. VSMCs were cultured in DMEM supplemented with 10% FBS. To induce osteogenic transdifferentiation, VSMCs between passages 3 and 7 were cultured in DMEM supplemented with 10 mM *β*-glycerophosphate. For transient transfection of miR-2861, miR-3960, anti-miR-2861, anti-miR-3960, and Runx2 siRNA, a mixture of each of them or their control with Lipofectamine 2000 (Invitrogen) was added to cells in 24-well plates at a density of 3 × 10^4^ cells per well for 48 hours.

### 2.2. Northern Blot

Total RNA was extracted from cells by Trizol reagent (Invitrogen). Northern blotting was performed as described previously [15]. 20 *μ*g of RNA was separated on a 15% urea polyacrylamide gel with 0.5x Tris borate-EDTA and transferred to a Hybond-N+ nylon membrane (Amersham Biosciences) using a semidry transfer cell (Bio-Rad). Hybridization was performed according to a standard protocol. ^32^P-labeled oligonucleotide probes complementary to the mature miR-2861 and miR-3960 were used in the hybridization. The probes for miR-2861, miR-3960, and U6 were 5′-CCGCCCGCCGCCAGGCCCC-3′, 5′-GCC­CCC­GCC­TCC­GCC­GCC­GCC-3′, and 5′-ATA­TGG­AAC­GCT­TCA­CGA­ATT-3′, respectively.

### 2.3. Western Blot

To detect Runx2, HDAC5, and Hoxa2 protein expression, Western blot analysis was performed as previously described [16, 17]. Briefly, equal amounts of protein (40 *μ*g/lane) were subjected to SDS gel electrophoresis and transferred to a polyvinylidene difluoride membrane (Amersham Pharmacia Biotech, GE Healthcare Biosciences, Pittsburgh, PA, USA). The membranes were incubated with primary antibodies against Runx2 (Santa Cruz), HDAC5 (Santa Cruz), Hoxa2 (Santa Cruz), and *β*-actin (Abcam) overnight at 4°C and then incubated with appropriate horseradish peroxidase-conjugated secondary antibodies (Santa Cruz) for 1 h at room temperature. The blots then were visualized with the chemiluminescent detection method using the SuperSignal West Pico Substrate (Pierce Biotechnology).

### 2.4. ALP Activity and Osteocalcin Secretion Assay

Cells were grown to confluence in 24-well plates. The cells were then washed with PBS and scraped into a solution containing 20 mM Tris-HCl, pH 8.0, and 150 mM NaCl and 1% Triton X-100, 0.02% NaN_3_, and 1 *μ*g/mL aprotinin. The lysates were homogenized; then alkaline phosphatase (ALP) activity was assayed by spectrophotometric measurement of p-nitrophenol release at 37°C. Osteocalcin released into the culture media was measured using a specific radioimmunoassay kit (DiaSorin). To normalize protein expression to total cellular protein, a fraction of the lysate solution was used in a Bradford protein assay.

### 2.5. qRT-PCR Analysis

Total RNA was isolated using the Trizol reagent (Invitrogen), and reverse transcription was performed using 1.0 mg of total RNA and the SuperScript II Kit (Invitrogen). qRT-PCR was performed using a Roche Molecular Light Cycler (Roche Molecular Biochemicals, Indianapolis, IN, USA). Amplification reactions were set up in 25 *μ*L reaction volumes containing SYBR Green PCR Master Mix (PE Applied Biosystems, Waltham, MA, USA), template cDNA, and amplification primers. The following primers were used: 5′-TGT­CAC­CGC­CAG­ATG­TTT­TG-3′, 5′-TGA­GCA­GAG­CCG­AGAC­ACA­G-3′; Hoxa2 forward, 5′-GTC­ACT­CTT­TGA­GCA­AGC­CC-3′; reverse, 5′-TAG­GCC­AGC­TCC­ACA­GTT­CT-3′; GAPDH sense, 5′-CAC­CAT­GGA­GAA­GGC­CGG­GG-3′; and antisense, 5′-GACGGACACATTGGGGGTAG-3′. PCR amplifications were performed, and the amplification data were analyzed using the sequence detector system software (PE Applied Biosystems). Relative quantification was calculated by normalizing the test crossing thresholds (Ct) with the GAPDH amplified control Ct.

### 2.6. Statistical Analyses

Data were presented as mean ± s.e.m. Comparisons were made using a one-way analysis of variance. All experiments were repeated at least three times, and representative experiments were shown. Differences were considered significant at *P* < 0.05.

## 3. Results

### 3.1. Increased Expression of miR-2861 and miR-3960 during the Osteogenic Transdifferentiation of VSMCs

**Figure 1:**
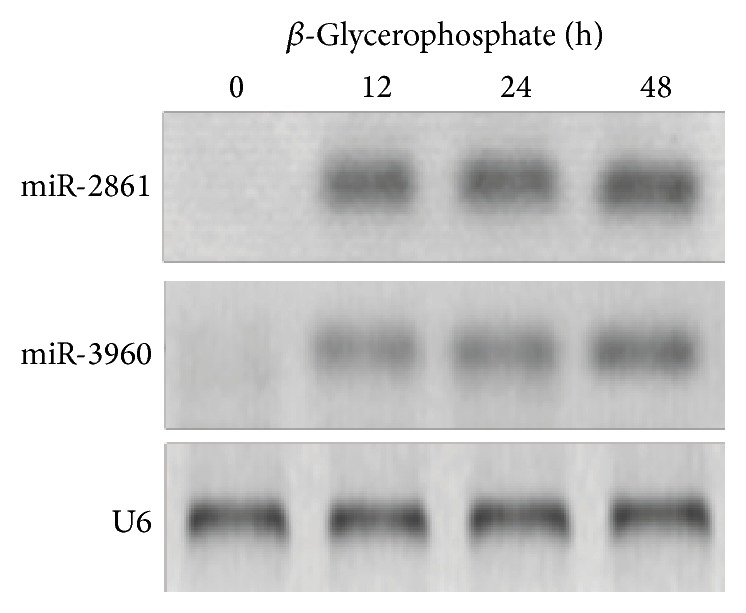
Increased expression of miR-2861 and miR-3960 during *β*-glycerophosphate-induced osteogenic transdifferentiation of VSMCs. Northern blot analysis of miR-2861 and miR-3960 expression in 10 mM *β*-glycerophosphate-treated VSMCs for the indicated times. U6 snRNA is used as a loading control.

We performed Northern blot analysis to examine the expression of miR-2861 and miR-3960 in VSMCs during *β*-glycerophosphate-induced osteogenesis. Both miR-2861 and miR-3960 could be detected after treatment with *β*-glycerophosphate for 12 h and increased with time throughout osteoblastic differentiation of VSMCs (Figure 1).

### 3.2. miR-2861 or miR-3960 Promotes Osteoblast Differentiation of Vascular Smooth Muscle Cells

**Figure 2:**
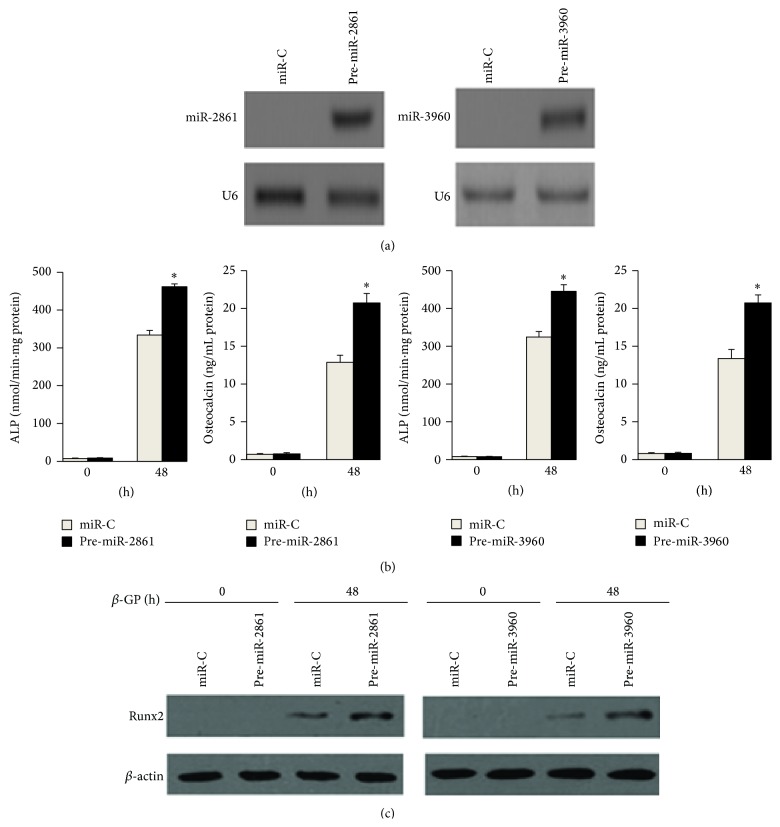
miR-2861 or miR-3960 promoted *β*-glycerophosphate-induced osteoblast differentiation of vascular smooth muscle cells. (a) Northern blot analysis of miR-2861 expression in VSMCs transfected with miR-C (control) or miR-2861 precursor (pre-miR-3861), miR-C, or pre-miR-3960. ((b) and (c)) ALP activity and osteocalcin secretion (b) and Western blot analysis of Runx2 protein expression (c) in 10 mM *β*-glycerophosphate- (*β*-GP-) treated VSMCs stably transfected with miR-C or pre-miR-2861, miR-C, or pre-miR-3960. Data are shown as mean ± s.e.m. ^*∗*^ *P* < 0.05.

**Figure 3:**
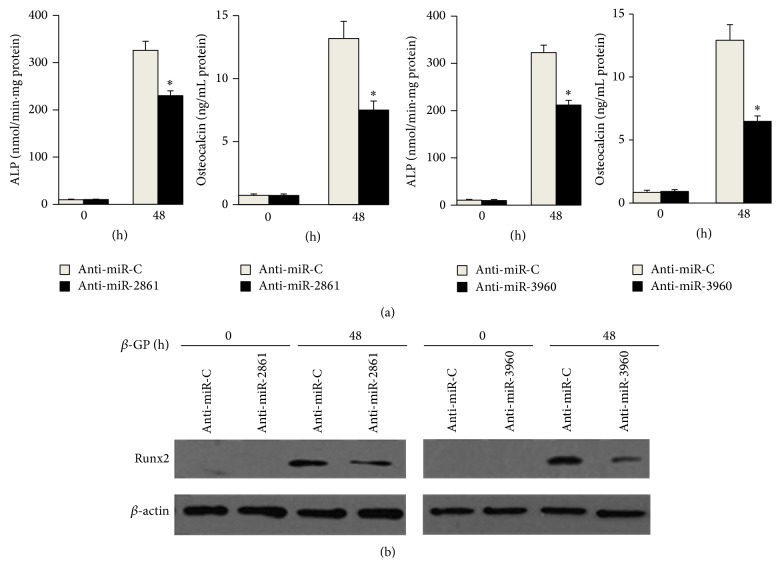
Inhibition of miR-2861 or miR-3960 inhibited *β*-glycerophosphate-induced osteoblast differentiation of vascular smooth muscle cells. ALP activity and osteocalcin secretion (a) and Western blot analysis of Runx2 protein expression (b) in 10 mM *β*-glycerophosphate- (*β*-GP-) treated VSMCs transfected with anti-miR-C (control) or anti-miR-2861, anti-miR-C, or anti-miR-3960. Data are shown as mean ± s.e.m. ^*∗*^ *P* < 0.05.

We next determined the role of miR-2861 or miR-3960 during osteogenic transdifferentiation of VSMCs by changing the functional levels of miR-2861 or miR-3960 in VSMCs. Northern blot data confirmed overexpression of miR-2861 and miR-3960 in VSMCs stably transfected with miR-2861 precursor and miR-3960 precursor, respectively (Figure 2(a)). The transfected VSMCs were treated with *β*-glycerophosphate for 48 h for inducing osteogenesis. Then, ALP activity, osteocalcin secretion, and Runx2 protein expression, as markers of osteoblast differentiation, were evaluated. The levels of ALP, osteocalcin, and Runx2 were all elevated in cells with overexpression of miR-2861 or miR-3960 (Figure 2(b)). These results indicated that overexpression of miR-2861 or miR-3960 promoted osteogenic transdifferentiation of VSMCs (Figures 2(b) and 2(c)). In contrast, knockdown of miR-2861 or miR-3960 reduced ALP, osteocalcin, and Runx2 levels induced by *β*-glycerophosphate (Figures 3(a) and 3(b)). All of these results suggest that both miR-2861 and miR-3960 act to promote osteogenic transdifferentiation of VSMCs.

### 3.3. miR-2861 and miR-3960 Posttranscriptionally Repress HDAC5 and Hoxa2 Expression

**Figure 4:**
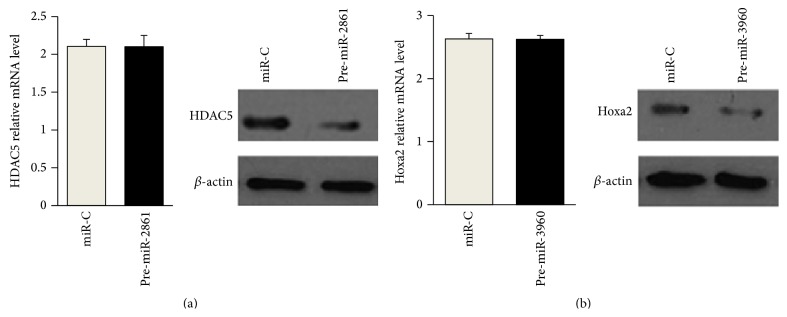
miR-2861 and miR-3960 posttranscriptionally repressed HDAC5 and Hoxa2 expression, respectively. ((a) and (b)) Quantitative RT-PCR analysis of mRNA levels of* HDAC5* and* Hoxa2 *(left) and Western blot analysis of protein levels of HDAC5 and Hoxa2 (right) in VSMCs transfected with miR-C (control) or pre-miR-3960 (a), miR-C, or miR-3960 (b). For quantitative RT-PCR analysis, mRNA levels normalized to GAPDH. Data are shown as means ± s.e.m. For Western blot analysis, *β*-actin acts as a loading control.

We previously identified that HDAC5 and Hoxa2 are the direct targets of miR-2861 and miR-3960, respectively, in a stromal cell line ST2 [13]. Here, we overexpressed miR-2861 or miR-3960 to directly identify their actions on HDAC5 or Hoxa2 in VSMCs. As expected, overexpression of miR-2861 or miR-3960 resulted in a decrease in the HDAC5 or Hoxa2 protein levels (Figures 4(a) and 4(b)). However, the HDAC5 or Hoxa2 mRNA levels were not affected (Figures 4(a) and 4(b)). These results revealed that miR-2861 and miR-3960 posttranscriptionally repressed HDAC5 and Hoxa2 protein expression by inhibiting mRNA translation in VSMCs.

### 3.4. Runx2 Stimulates miR-2861 and miR-3960 Expression in Vascular Smooth Muscle

**Figure 5:**
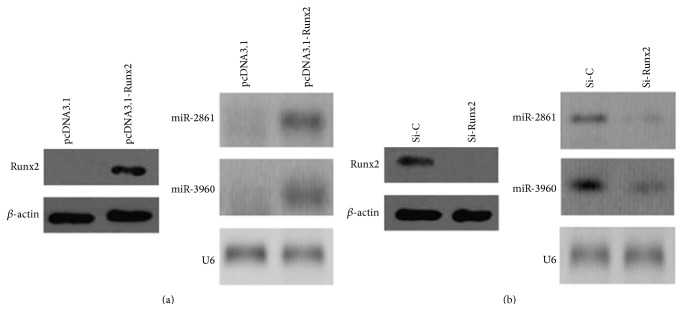
Runx2 stimulates miR-2861 and miR-3960 expression in vascular smooth muscle cells. (a) Western blot analysis of Runx2 protein levels (left) and Northern blot analysis of miR-2861 and miR-3960 levels (right) in VSMCs transfected with pcDNA3.1 vector control or Runx2 pcDNA3.1 vector (*pcDNA3.1-Runx2*) for 48 h. (b) Western blot analysis of Runx2 protein levels (left) and Northern blot analysis of miR-2861 and miR-3960 levels (right) in VSMCs transfected with siRNA control (*si-C*) or si-Runx2 and then cultured with *β*-glycerophosphate for 48 h. For Western blot analysis, *β*-actin acts as a loading control. For Northern blot analysis, U6 snRNA acts as a loading control.

We previously demonstrated that Runx2 directly induces the expression of the miR-3960/miR-2861 cluster by binding to the putative binding site of its promoter in ST2 cells [13]. Here, in order to identify the direct impact of Runx2 on miR-2861 and miR-3960 gene expression in VSMCs, we changed the functional levels of Runx2. First, we used a pcDNA-driven expression vector to overexpress Runx2 protein levels, as confirmed by Western blot (Figure 5(a)). The results showed that Runx2 overexpression could induce miR-2861 and miR-3960 expression in VSMCs (Figure 5(a)). Furthermore, we transfected siRNA designed against Runx2 into *β*-glycerophosphate-induced VSMCs to specifically silence Runx2 expression. Western blot analysis revealed that si-Runx2 blocked Runx2 expression compared with the control (Figure 5(b)). The expression of miR-3960 and miR-2861 was inhibited by the knockdown of Runx2 (Figure 5(b)). Thus, these results suggest a direct stimulatory action of Runx2 on the expression of miR-2861 and miR-3960 in VSMCs.

## 4. Discussion

In our previous study, we cloned both miR-2861 and miR-3960 and identified that miR-2861 induces osteoblast differentiation by repressing HDAC5, an enhancer of Runx2 degradation, and miR-3960 induces osteoblast differentiation by repressing Hoxa2, a repressor of Runx2 expression [13, 15]. miR-2861 binds to the coding domain sequence (CDS) of* HDAC5* mRNA with complementarity to the miR-2861 seed region and posttranscriptionally represses HDAC5 protein expression by inhibiting mRNA translation and not by mRNA degradation [15]. Similarly, miR-3960 directly targets* Hoxa2* by specifically binding with the target CDS of* Hoxa2* and represses Hoxa2 expression at the posttranscriptional level [13]. Thus, miR-3960 and miR-2861 can coregulate the Runx2 during osteoblast differentiation. In turn, Runx2 binds to the miR-2861/miR-3960 cluster promoter to transcriptionally induce the expression of miR-2861 and miR-3960 [13]. Runx2/miR-3960/miR-2861 is a critical positive feedback loop for osteoblast differentiation.

Recently, several miRNAs have been reported to be involved in osteogenic transdifferentiation and calcification of VSMCs [18–22]. It has been reported that miR-221 and miR-222 increase runt-related transcription factor 2 (Runx2) expression and calcium deposition in VSMCs [18]. On the contrary, miR-204, miR-133a, and miR-125b suppress osteoblastic differentiation of VSMCs by inhibiting Runx2 protein expression, whereas miR-204, miR-133a, and miR-125b inhibition enhance osteoblastic differentiation of VSMCs [19–22]. In this study, we show that Runx2/miR-3960/miR-2861 is also responsible for stimulating osteogenic transdifferentiation of VSMCs. At first, we demonstrated that the expression of both miR-2861 and miR-3960 was significantly increased during *β*-glycerophosphate-induced osteogenic transdifferentiation of VSMCs, indicating that miR-2861 and miR-3960 might play a role in vascular calcification. To prove whether miR-2861 and miR-3960 are directly associated with the osteogenic transdifferentiation of VSMCs, we evaluated the effect of miR-2861 and miR-3960 in the process of *β*-glycerophosphate-induced osteogenic transdifferentiation of VSMCs. Runx2 has been identified as a “master transcription factor” simulating osteogenic transdifferentiation of precursor cells [4, 23]. It plays an essential role in the osteogenic transdifferentiation of VSMCs [24, 25]. Knockdown of Runx2 significantly inhibits the ALP expression and the calcification in the senescent VSMCs [26]. ALP is a prophase (proliferative phase) marker of osteoblast differentiation. Osteocalcin is a metaphase (bone matrix formation period) marker of osteoblast differentiation. Both miR-2861 and miR-3960 promoted osteogenic transdifferentiation of VSMCs, as indicated by increased levels of ALP activity, osteocalcin secretion, and Runx2 protein expression.

HDAC5 is an enhancer of Runx2 degradation. It deacetylates Runx2, allowing the protein to undergo Smurf-mediated degradation, and inhibition of HDAC5 increases Runx2 acetylation [27, 28]. Hoxa2, a member of the Hox homeodomain family of transcription factors that regulate skeletal patterning, is found to control Runx2 expression and is required in skeletal morphogenesis [29].* Hoxa2* ^−/−^ mice show an upregulation of the Runx2 level, and Hoxa2 inhibits bone formation by repressing Runx2 expression [29]. In our previous study, ST2 cells transfected with a Hoxa2 expression vector showed reduced Runx2 expression and ALP activity, confirming that Hoxa2 can suppress Runx2 expression and further inhibits osteoblast differentiation [13]. In this study, we detected HDAC5 expression in VSMCs, inconsistent with several previous papers [29–32]. Moreover, we identified Hoxa2 expression in VSMCs. Thus, miR-2861 and miR-3960 directly target* HDAC5* and* Hoxa2*, respectively, to increase Runx2 levels in VSMCs.

Together, our study shows that Runx2/miR-3960/miR-2861 positive feedback loop plays an important role in osteogenic transdifferentiation of VSMCs and contributes to vascular calcification.
